# HiJAK’d Signaling; the STAT3 Paradox in Senescence and Cancer Progression

**DOI:** 10.3390/cancers6020741

**Published:** 2014-03-26

**Authors:** Damian J. Junk, Benjamin L. Bryson, Mark W. Jackson

**Affiliations:** Department of Pathology, Case Western Reserve University, Case Comprehensive Cancer Center, 2103 Cornell Road, WRB 3-134, Cleveland, OH 44106, USA; E-Mails: damian.junk@case.edu (D.J.J.); blb74@case.edu (B.L.B.)

**Keywords:** oncostatin M, gp130, STAT3, cytokine, senescence, transformation, inflammation

## Abstract

Clinical and epidemiological data have associated chronic inflammation with cancer progression. Most tumors show evidence of infiltrating immune and inflammatory cells, and chronic inflammatory disorders are known to increase the overall risk of cancer development. While immune cells are often observed in early hyperplastic lesions *in vivo*, there remains debate over whether these immune cells and the cytokines they produce in the developing hyperplastic microenvironment act to inhibit or facilitate tumor development. The interleukin-6 (IL-6) family of cytokines, which includes IL-6 and oncostatin M (OSM), among others (LIF, CT-1, CNTF, and CLC), are secreted by immune cells, stromal cells, and epithelial cells, and regulate diverse biological processes. Each of the IL-6 family cytokines signals through a distinct receptor complex, yet each receptor complex uses a shared gp130 subunit, which is critical for signal transduction following cytokine binding. Activation of gp130 results in the activation of Signal Transducer and Activator of Transcription 3 (STAT3), and the Mitogen-Activated Protein Kinase (MAPK) and Phosphatidylinositol 3-Kinase (PI3K) signaling cascades. Tumor suppressive signaling can often be observed in normal cells following prolonged STAT3 activation. However, there is mounting evidence that the IL-6 family cytokines can contribute to later stages of tumor progression in many ways. Here we will review how the microenvironmental IL-6 family cytokine OSM influences each stage of the transformation process. We discuss the intrinsic adaptations a developing cancer cell must make in order to tolerate and circumvent OSM-mediated growth suppression, as well as the OSM effectors that are hijacked during tumor expansion and metastasis. We propose that combining current therapies with new ones that suppress the signals generated from the tumor microenvironment will significantly impact an oncologist’s ability to treat cancer.

## 1. The Inflammatory Microenvironment; from Cancer Suppression to Cancer Progression

Clinical and epidemiological data have associated chronic inflammation with cancer progression [1]. Most tumors have extensive evidence of infiltrating immune and inflammatory cells, and chronic inflammatory disorders are known to increase the overall risk of cancer development [2,3]. For example, persistent inflammation produced due to infection can promote GI cancers (*H. pylori*), hepatocellular carcinoma (hepatitis B and C), and genital cancer (papilloma viruses). In addition, persistent inflammation that is not due to infection can promote colon, esophageal, pancreatic, and gallbladder cancers [4]. Immune cells found in the tumor microenvironment include macrophages, lymphocytes, neutrophils, eosinophils, and dendritic cells [2,3]. The infiltration of macrophages into a tumor has been correlated with poor prognosis, often due to the stimulation of blood vessel formation and the chronic cycle of tissue damage and proliferative repair created by the persistent presence of inflammatory immune cells [5]. In addition to infiltrating immune cells, cancer cells can influence neighboring fibroblasts to incite the abnormal production of inflammatory cytokines [6,7]. Cancer cells, themselves, can secrete inflammatory cytokines that enhance immune cell infiltration and shape the tumor microenvironment. However, there remains debate over whether the inflammatory cytokines act solely to promote tumor development, especially during the earliest stages of hyperplastic growth, or whether certain cells and cytokines can actually suppress tumor development [2].

**Figure 1:**
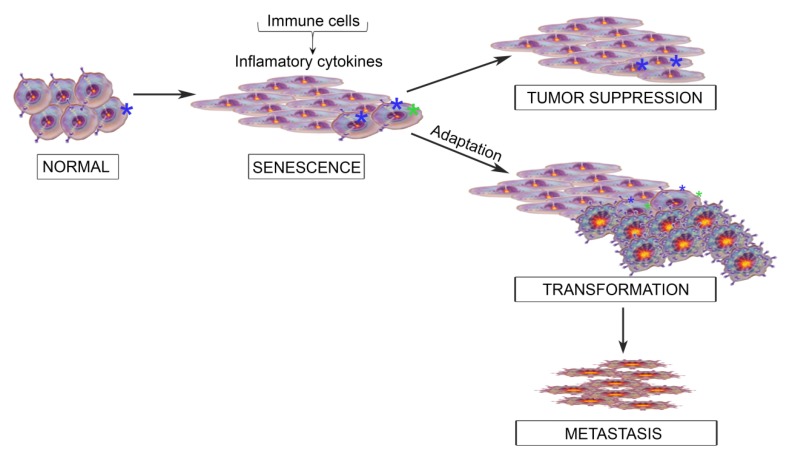
Modeling the role of inflammatory cytokines in cellular transformation. Schematic illustrates how immune cells can infiltrate into normal tissue following the acquisition of transforming genetic events in epithelial cells (denoted by *). Recruitment of immune cells and the inflammatory cytokines they produce can either (1) permanently suppress the growth of the dysregulated cells, resulting in tumor suppression or (2) apply a selective pressure to the dysregulated cells, resulting in adaptive changes to escape the senescence-inducing signals.

We propose that at the earliest stage of cancer development, when a cell has acquired a genetic change that promotes inappropriate proliferation, the initial immune system response engages the aberrant cells in a biological game of chess. Cytokines secreted as part of the immune response slow or stop the growth of early hyperplastic lesions. In order to continue on its path towards transformation, a developing cancer cell must adapt to bypass the immune-mediated growth suppression. As the evolving chess game unfolds, the growth of the developing cancer cell may be permanently thwarted by the immune response, as tumor suppression prevails. Alternatively, if the developing cancer cells can circumvent the tumor suppressive response engaged by these inflammatory cytokines, then the transformation process would continue. As transformation progresses, cytokine signaling may even contribute to the acquisition of more aggressive tumor phenotypes (Figure 1). The interleukin-6 (IL-6) family of cytokines is at the forefront of the debate over the role of inflammatory cytokines in tumor suppression or progression [8,9]. The focus of this review is to discuss these opposing roles of persistent IL-6 family signaling, with emphasis on oncostatin M (OSM) exposure and the resulting Signal Transducer and Activator of Transcription 3 (STAT3) hyperactivation.

## 2. The IL6 Family of Cytokines

**Figure 2:**
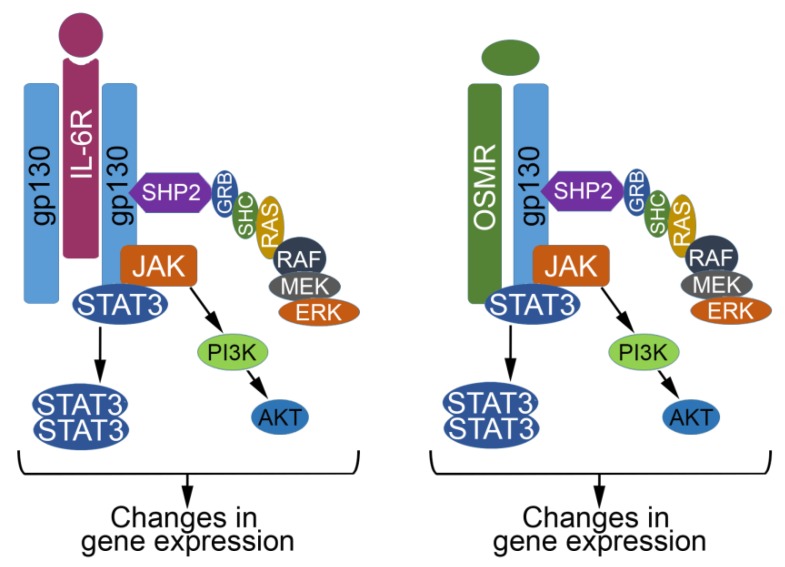
Signaling from the gp130 receptor in response to IL-6 and OSM. In the presence of IL-6 or OSM, Activation of gp130 results in the activation of Signal Transducer and Activator of Transcription 3 (STAT3), and the Mitogen-Activated Protein Kinase (MAPK) and Phosphatidylinositol 3-Kinase (PI3K) signaling cascades as shown. Phosphorylated STAT3 dimerizes, translocates to the nucleus, and promotes transcription of target genes.

The IL-6 family of cytokines, which includes IL-6, OSM, leukemia inhibitory factor (LIF), cardiotrophin-1 (CT-1), and ciliary neurotrophic factor (CNTF) are secreted by immune cells, stromal cells, and epithelial cells, and regulate diverse biological processes [9,10,11]. Each of the IL-6 family cytokines signals through a distinct receptor complex, yet each receptor complex uses a common glycoprotein 130 (gp130) subunit, which is critical for signal transduction following cytokine binding (Figure 2; [11]). Ligand activation of gp130 results in the recruitment of Janus Kinases (JAKs) and phosphorylation of STAT3. Phosphorylated STAT3 dimerizes, translocates to the nucleus, and promotes transcription of target genes. Many genes targeted by phosphorylated STAT3 are key regulators of cell proliferation and survival, such as c-MYC, JunB, Cyclin D1, Mcl-1, Bcl-xl, Survivin, Pim1, and Pim2 [12,13]. Following cytokine-induced STAT3 activation, suppressor of cytokine signaling 3 (SOCS3) and protein inhibitor of activated STAT3 (PIAS3) act as negative regulators to prevent the excessive expression of downstream STAT3-target genes. Activation of gp130 can also stimulate Mitogen-Activated Protein Kinase (MAPK), and Phosphatidylinositol 3-Kinase (PI3K) signaling cascades [11]. Given that STAT3, MAPK, and PI3K signaling have diverse roles in normal development, it is not surprising that gp130 activation may play a paradoxical role in tumor suppression [11]. OSM is an excellent example of an inflammatory cytokine found in the tumor microenvironment that has tumor suppressive properties in specific contexts. Below, we discuss what is known about how OSM engages JAK/STAT3 signaling to engage tumor suppressive signaling, and paradoxically to promote tumor progression, metastasis, and cancer stem cell expansion.

## 3. When OSM and JAK/STAT Signaling Behave; Acting as a Tumor Suppressor

OSM was originally described as a novel, biological cancer therapy because of its ability to inhibit the growth of melanoma cells [14]. Additional studies have confirmed the anti-proliferative activity of OSM in select breast, lung, and glioma cell lines [10]. In normal human mammary epithelial cells (HMEC), persistent exposure to OSM engages a senescent phenotype similar to the Oncogene-Induced Senescence (OIS) originally described following expression of dysregulated RAS in normal cells [15,16]. Dysregulated expression or activation of a number of oncogenes (RAF, AKT, E2F, MOS, CyclinE, CDC6) or loss of tumor suppressors (PTEN or NF1) has now been reported to engage OIS programs [17]. It is now clear that OIS is a critical tumor suppressive barrier *in vivo*. For example, the presence of senescent cells in benign or early stage cancers, but not advanced cancers, argues that OIS serves as an early tumor suppressive barrier that needs to be dismantled for full oncogenic progression [18,19,20,21,22,23,24,25,26]. These data support the argument that OIS is a physiologically relevant tumor-suppressive mechanism. Defining unique tumor suppressive mechanisms engaged in normal cells the engage OIS may lead to the development of targeted therapies that reengage senescence and enhance outcome and overall survival of cancer patients.

A large number of studies provide evidence that early stage tumor formation is halted quite frequently. For example, recent studies have identified pre-malignant breast lesions in more than half of women who underwent elective mammaplasty reduction surgery, while only one-in-eight women progress to clinical breast cancer in their lifetime [27,28]. Similar observations have been made during autopsies performed on women with no medical history of breast cancer, in which 39% of women in their forties had undiagnosed breast cancer [29]. Again, given the one-in-eight lifetime risk of cancer, many breast cancers never fully progress to a stage that will be diagnosed. Occult tumors such as those observed in the breast are frequently identified in many other tissues as well, including prostate, thyroid, lung, pancreas, and kidney (among others) upon autopsy for death unrelated to cancer [30]. The presence of stalled lesions or occult tumors indicate that proliferation is being constrained by tumor suppressive senescence, which may include JAK/STAT3 signaling. An involvement of JAK/STAT3 in the tumor suppressive senescence induced in developing breast lesions may have adverse consequences for the use of JAK/STAT3 signaling inhibitors, such as approved drugs tofacitinib or ruxolitinib, or drugs in clinical trials such as baricitinib, lestaurtinib, pacritinib, or TG101348. The use of such inhibitors may prevent STAT3-mediated senescence and allow the outgrowth of dormant or occult tumors, thereby promoting cancer progression.

## 4. OSM Turns to the Dark Side; Acting as a Tumor Promoter

Despite the many studies implicating OSM as a suppressor of normal cell and select tumor cell proliferation, OSM has also been implicated, paradoxically, in cancer progression. Expression of OSM is significantly elevated in the breast, prostate, cervical, and ovarian tumor microenvironments, and several studies provide evidence implicating elevated OSM in metastasis and increased risk of tumor recurrence [15,31,32,33]. First, in addition to the increased presence of OSM in the tumor microenvironment, cancer cells can also express high levels of OSM receptor (OSMR), which is associated with adverse clinical outcomes [31,32,33]. Importantly, the highly aggressive basal-like or triple-negative breast cancer subtypes express markedly higher levels of OSMR when compared to the less aggressive luminal subtype [34]. Second, not only do certain cells fail to undergo a proliferative arrest upon challenge with OSM, but OSM actually stimulates the proliferation of diverse cells, including Kaposi’s sarcoma cells, dermal fibroblasts, and plasmacytoma cells. Furthermore, OSM can induce increased migration and invasiveness of select breast cancer cell lines, implicating OSM in metastasis, which is discussed below [10,35,36,37]. Finally, exposure of peritoneal and bone marrow-derived macrophages to the chemotherapy drug cisplatin induces significant OSM secretion [38,39]. When taken together, there is significant evidence implicating microenvironmental OSM as not only an oncogenic signal, but also as a potential contributor to therapeutic resistance and tumor recurrence. 

## 5. Beyond Transformation: OSM and JAK/STAT3 Signaling in Metastasis and Cancer Stem Cell Phenotypes

The spread of a primary tumor to distant secondary sites, metastasis, is the cause of 90% of death due to cancer, yet our understanding of this complex problem remains limited [40]. Epithelial-mesenchymal transition (EMT) is an essential developmental process during embryogenesis that creates mesodern and the neural tube [41]. EMT involves the loss of epithelial traits and acquisition of mesenchymal traits. During development, cells that have undergone EMT are able to migrate to specific sites in the embryo, where they then differentiate to form distinct structures and organs. Likewise, during the initiation of metastasis, select epithelial tumor cells undergo EMT resulting in the loss of cell-cell adhesion and the acquisition of a mesenchymal phenotype with increased motility and invasiveness [41,42,43]. Following EMT, the mesenchymal cancer cell has the ability to migrate away from the primary tumor and to invade adjacent tissue. Molecular changes intrinsic to a cancer cell and resulting from signaling cascades activated by exogenous cytokines arising from the tumor microenvironment (TME), drive EMT and metastasis [41,44,45,46,47,48,49,50,51,52,53,54,55].

High levels of OSM observed in primary tumors are now being implicated in more than just dysregulated proliferation. Recent studies suggest that exogenous OSM exposure induces EMT linking OSM-STAT3 signaling to metastasis [34,35,36,37,56]. Increased levels of OSM protein are reported to be concentrated at the invasive edges of breast tumors suggesting a role for OSM in tumor invasion [56,57]. OSM is secreted autonomously by activated tumor-associated macrophages (TAMs), or secreted by the paracrine stimulation of neutrophils and TAMs by nearby breast cancer cells [58]. TAMs are localized at the invasive edges of breast tumors and have been known to promote cancer cell invasion by secreting epidermal growth factor (EGF; [56]). Importantly, OSM is co-expressed with EGF by TAMs, and the levels of secreted OSM are strongly correlated with levels of secreted EGF in breast cancer patients [56]. Furthermore, TAM-derived EGF and OSM cooperatively promoted cancer cell migration, implying that OSM collaborates with other soluble factors in the TME to facilitate cancer development and progression. Moreover, OSMR has the ability to bind to non-cytokine receptor subunits and has been demonstrated to interact with another EGFR family member, HER2/ErbB-2, which is often elevated in metastatic breast tumors [59].

Seminal work demonstrated that EMT generates mesenchymal cell populations with cancer stem-cell (CSC) phenotypes [55]. Unlike their specialized progeny, stem cells are capable of long-term self-renewal, even after prolonged periods of inactivity [60]. Stem cells often reside within specialized niches, and can be maintained in a non-proliferative state by their microenvironment [61]. In response to proper stimuli, stem cells can divide symmetrically to produce more stem cells (self-renewal) or asymmetrically to generate specialized progeny (differentiation). CSC retain many of the properties of their normal stem-cell counterparts [62]. CSC generate heterogeneous tumors from a limited number of starting cells. CSC pools within tumors are maintained by self-renewal, while the bulk tumor is created by the generation of more differentiated cancer cells. CSC are refractory to conventional cancer therapies that typically target the highly proliferative cells of the bulk tumor [63]. Therefore, CSC survive most therapeutic interventions and seed tumor recurrence after the bulk tumor has been reduced. Thus, CSC are a highly desirable target for the development of novel effective cancer therapies.

The effect of OSM on CSC is contradictory. OSM causes the differentiation of liver cancer cells, making them more susceptible to 5-fluoro-uracil [64]. OSM can also induce cell differentiation in certain breast cancers [65]. However, OSM promotes the growth and generation of CSC in Ewing’s sarcoma and causes EMT and the generation of CSC in tubular epithelial and breast cancer cells [66,67,68]. Interestingly, a novel CSC specific therapy, was shown to induce cell death by inhibiting STAT3 [69]. Further studies implicate STAT3 activation in the generation of CSC properties in breast cancer, liver cancer, and glioblastoma cells [70,71,72,73]. OSMR expression is elevated in the more aggressive basal-like breast cancer subtype, in comparison to the less aggressive luminal subtype [34]. The elevated OSMR expression coincides with acquisition of the CSC cell surface marker CD44, and parallels the elevated expression of epidermal growth factor receptor and Transforming Growth Factor-β receptor type I, two receptor kinases with established roles in cancer development and progression [34].

Elevated levels of OSM mRNA isolated from micro-dissected tumor stroma are associated with an increased risk of tumor recurrence, highly aggressive metastatic cancers, and poor patient prognosis [15,34,74]. Furthermore, cross-talk between tumor stroma and tumor epithelial cells that promotes CSC phenotypes required STAT3 signaling within the tumor associated fibroblasts [75]. Together, these results suggest that exogenous OSM signaling through STAT3 may be a major contributor to the CSC phenotypes of tumors, and implicate the microenvironment as a critical determinant of tumor seeding and recurrence. Therefore, targeting OSM emanating from the TME may be an effective strategy to target CSC and inhibit tumor recurrence.

## 6. Integrating the Paradoxical Roles for OSM into a Model of Tumor Progression

**Figure 3:**
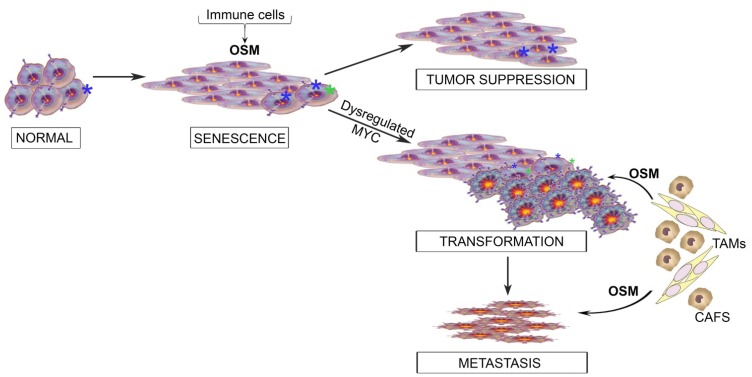
Modeling the role of OSM in cellular transformation. Schematic illustrates how immune cells can infiltrate into normal tissue following the acquisition of transforming genetic events in epithelial cells (denoted by *). Recruitment of immune cells results in the production of OSM, which can suppress the growth of the dysregulated cells, resulting in tumor suppression. Alternatively, the continued presence of OSM creates a selective pressure on the dysregulated cells, resulting in adaptive changes, such as increased MYC expression, and escape from OSM-mediated senescence. Sustained proliferation results in full transformation, and bi-directional signaling between cancer cells and stromal cells creates an evolving tumor microenvironment (TME). The TME may also support the conversion of epithelial cells into cancer stem cells (CSC) with increased invasive and metastatic capabilities.

Recent studies from our laboratory begin to address the disparate results reported with OSM in breast cancer. Using an isogenic, step-wise HMEC transformation model, the persistent OSM-mediated activation of JAK/STAT3 signaling was examined across numerous, isogenic HMEC derivatives representing distinct stages of the transformation process [15]. In normal HMEC, and derivatives lacking the tumor suppressors p16INK4a and p53, OSM signaling induces a STAT3-dependent tumor suppressive senescence which is engaged and enforced by the transcriptional suppression of the c-MYC gene. We propose that early breast hyperplasia engages microenvironmental responses from infiltrating immune cells, or other stromal components that lead to persistent JAK/STAT3-signaling and a tumor suppressive arrest (Figure 3). The senescent cells, themselves, may then secrete additional cytokines that lead to a wide-spread senescence through a bystander effect [76,77,78,79]. The prototype molecule for OIS studies is mutant RAS. While RAS is a powerful oncogene in many cancers, its expression in normal cells creates a strong, irreversible senescence response [80]. Studies of RAS-mediated OIS demonstrated that constitutive c-MYC expression could prevent OIS and then collaborate with additional RAS-driven signals to promote a transformed phenotype. c-MYC is one of the most frequently de-regulated oncogenes and is commonly up-regulated in many human cancer types [81]. The c-MYC gene encodes the oncoprotein, c-MYC, a potent transcription factor involved in regulating the expression of up to 15% of the entire human genome. Dysregulated c-MYC promotes cell growth and proliferation by up-regulating the expression of genes involved in driving cell cycle progression, such as cdc25A, cyclin D1, cyclin E, and cyclin A. Dysregulated c-MYC also prevents cell cycle arrest by repressing expression of the cyclin-dependent kinase inhibitor, p15INK4b [82]. Importantly, dysregulated c-MYC greatly abrogates the senescence response induced by OSM, arguing a common link between OSM-mediated OIS and RAS-mediated OIS [15]. Importantly, once the OSM OIS response is dismantled by dysregulated MYC expression, additional signaling emanating from OSMR activation, which includes pro-oncogenic STAT3, PI3K, and MAPK effectors, can drive the expansion of premalignant cells.

The ability of OSM to induce senescence in one context and proliferation or increased aggressiveness in another context is similar to the paradoxical signaling observed with Transforming Growth Factor Beta (TGF-β) [83]. The differential response of normal, hyperplastic, and transformed cells to the presence of TGF beta can be explained by the diverse signals that can emanate from the TGF-β receptor [84]. Importantly, the identification of intrinsic genetic events that can cooperate with OSM in the tumor microenvironment to consistently drive transformation is providing information on how important tumor suppressive barriers can be overcome in normal cells as they progress towards malignancy. There is considerable support for the role of OSM as a growth suppressor during distinct phases of the transformation process, and in distinct cell types. For example, comparative analysis of normal, primary human bronchial epithelial cells (HBEC) and abnormal, premalignant HBECs demonstrate that OSM is capable of suppressing the proliferation of normal HBEC [85]. However, the ability of OSM to suppress the proliferation of abnormal, premalignant HBECs is significantly compromised, even at early stages of the transformation process. Isolation of pulmonary macrophages identified OSM and IL-6 among the secreted cytokines. Together these finding indicate that even during the earliest stage of HBEC transformation, cells become refractory to cytokines produced by infiltrating immune cells.

In other tissues and cancer types, the senescence response engaged by persistent JAK/STAT3 signaling may be more difficult to hijack. Determining which tumors retain a response to IL-6 family cytokines may provide a useful avenue for cancer therapy. For example, patients with melanoma have an increased relapse-free survival if short-term cultures of their cancer cells retained a sensitivity to OSM and/or IL-6 [86]. The increase in relapse-free survival in these patients was attributed to the anti-proliferative effect of OSM, not induction of apoptosis. Senescence is increasingly being recognized as a major tumor-suppressive barrier *in vivo*, and is now being targeted to develop novel “pro-senescence” therapies [87,88,89]. Depending upon how senescence signaling is dismantled during tumorgenesis, the components necessary to engage senescence may simply be masked rather than absent. Thus, pro-senescence therapies aim to reactivate these masked senescence components to reengage the hidden senescence response. Defining unique tumor suppressive mechanisms engaged by pro-senescence OSM signaling may lead to the development of targeted therapies that reengage senescence and enhance outcome and overall survival of cancer patients.

## 7. Conclusions

A greater understanding of how cancer cells hijack the OSM produced in the microenvironment near normal, premalignant, and fully transformed epithelial cells and block the initial JAK/STAT3-mediated tumor suppression signaling in favor of transformation and increased tumor heterogeneity is highly significant. We suggest that the growth suppression engaged in normal cells and tissues by the recruitment of immune cells exposes the developing hyperplasia to OSM, which engages a JAK/STAT3-mediated tumor suppressive response. Over time, the tumor suppressive signaling can be dismantled, perhaps by dysregulated MYC expression, allowing progression of the transformation process. Bypassing the growth suppressive signaling leaves gp130-activated signals (STAT3, MAPK, and PI3K/AKT) intact to now serve as downstream effectors to drive aggressive OSM-mediated tumor phenotypes. Defining the molecular mechanisms that extracellular factors such as OSM use to drive cancer development and progression (or the processes that mask the pro-senescent outcome) may facilitate the development of targeted therapies capable of reengaging hidden senescence signals to suppress tumor growth and extend patients’ lives.
